# Fully quantitative mapping of abnormal aortic velocity and wall shear stress direction in patients with bicuspid aortic valves and repaired coarctation using 4D flow cardiovascular magnetic resonance

**DOI:** 10.1186/s12968-020-00703-2

**Published:** 2021-02-15

**Authors:** Pim van Ooij, Emile S. Farag, Carmen P. S. Blanken, Aart J. Nederveen, Maarten Groenink, R. Nils Planken, S. Matthijs Boekholdt

**Affiliations:** 1 Department of Radiology and Nuclear Medicine, Amsterdam University Medical Center, location AMC, Meibergdreef 9, 1105 AZ Amsterdam, The Netherlands; 2 Department of Cardiothoracic Surgery, Amsterdam University Medical Center, location AMC, Amsterdam, The Netherlands; 3 Department of Cardiology, Amsterdam University Medical Center, location AMC, Amsterdam, The Netherlands

## Abstract

**Background:**

Helices and vortices in thoracic aortic blood flow measured with 4D flow cardiovascular magnetic resonance (CMR) have been associated with aortic dilation and aneurysms. Current approaches are semi-quantitative or when fully quantitative based on 2D plane placement. In this study, we present a fully quantitative and three-dimensional approach to map and quantify abnormal velocity and wall shear stress (WSS) at peak systole in patients with a bicuspid aortic valve (BAV) of which 52% had a repaired coarctation.

**Methods:**

4D flow CMR was performed in 48 patients with BAV and in 25 healthy subjects at a spatiotemporal resolution of 2.5 × 2.5 × 2.5mm^3^/ ~ 42 ms and TE/TR/FA of 2.1 ms/3.4 ms/8° with k-t Principal Component Analysis factor *R* = 8. A 3D average of velocity and WSS direction was created for the normal subjects. Comparing BAV patient data with the 3D average map and selecting voxels deviating between 60° and 120° and > 120° yielded 3D maps and volume (in cm^3^) and surface (in cm^2^) quantification of abnormally directed velocity and WSS, respectively. Linear regression with Bonferroni corrected significance of P < 0.0125 was used to compare abnormally directed velocity volume and WSS surface in the ascending aorta with qualitative helicity and vorticity scores, with local normalized helicity (LNH) and quantitative vorticity and with patient characteristics.

**Results:**

The velocity volumes > 120° correlated moderately with the vorticity scores (R ~ 0.50, P < 0.001 for both observers). For WSS surface these results were similar. The velocity volumes between 60° and 120° correlated moderately with LNH (R = 0.66) but the velocity volumes > 120° did not correlate with quantitative vorticity. For abnormal velocity and WSS deviating between 60° and 120°, moderate correlations were found with aortic diameters (R = 0.50–0.70). For abnormal velocity and WSS deviating > 120°, additional moderate correlations were found with age and with peak velocity (stenosis severity) and a weak correlation with gender. Ensemble maps showed that more than 60% of the patients had abnormally directed velocity and WSS. Additionally, abnormally directed velocity and WSS was higher in the proximal descending aorta in the patients with repaired coarctation than in the patients where coarctation was never present.

**Conclusion:**

The possibility to reveal directional abnormalities of velocity and WSS in 3D provides a new tool for hemodynamic characterization in BAV disease.

## Introduction

In the past decade, volumetric and time-resolved velocity and wall shear stress (WSS) measurements in the thoracic aorta with 4D flow cardiovascular magnetic resonance (CMR) have provided important insights in the relationship between altered aortic hemodynamics as a consequence of a bicuspid aortic valve (BAV) and concomitant aortic dilation [1–6]. Crucial findings that supported the link between altered hemodynamics and aortopathy were the association of 4D flow CMR-derived WSS with elastic fiber degeneration, extracellular matrix dysregulation and aortic wall stiffness [7, 8]. Consequentially, 4D flow CMR is now recommended in recent guidelines as an important tool that provides added criteria for surveillance, indication for surgery and follow-up in BAV-related aortopathy [9].

The notion of altered hemodynamics does not only imply that the magnitude of velocity and WSS is different in patients compared to people with a trileaflet aortic valve, but also that the direction of blood flow and WSS differs. The formation of helices and vortices in aortic blood flow has gained clinical interest since a possible relation between abnormally directed blood flow and aortic dilation exists [10–12]. As such, the formation of helical and vortical flow as visualized by 4D flow CMR in thoracic aortic aneurysms and dilated aortas has been extensively described [10–12]. To relate altered flow to aortic diameters, the assessment of helical and vortical flow has gradually changed from semi-quantitative scoring of velocity streamline directions [12–15] to fully quantitative approaches [16–23]. In addition, helical flow in the descending aorta distal to repaired coarctation has been implicated in the development of hypertension [24]. However, these approaches rely on placement of analysis planes and manually chosen thresholds, or are dependent on streamline or pathline creation.

Furthermore, since vascular disease is promoted by low and oscillatory WSS [25, 26], the multidirectionality of WSS has been extensively investigated by comparison with the curvature of the aorta [19, 22, 27]. However, the comparison of pathological axial and circumferential WSS with the normal situation may be confounded by an altered aortic shape in BAV disease [14].

In this study a novel methodology is presented to visualize and quantify peak systolic 3D abnormal direction of velocity and WSS in patients with BAV and patients with BAV and repaired coarctation based on a comparison with 3D average velocity and WSS vector maps of normal subjects. We hypothesize that the extent of abnormally directed velocity and WSS (1) in the ascending aorta compares with helicity and vorticity scoring of velocity streamlines, (2) in the descending aorta is larger in patients with repaired coarctation than without, (3) agrees well with local normalized helicity [21] and vorticity [28] quantification and (4) in the ascending aorta correlates with aortic diameters, aortic valve stenosis severity and aortic regurgitation severity. Furthermore, ensemble maps are presented to highlight the location and incidence of abnormally directed velocity and WSS in BAV disease.

## Methods

### Subject enrollment

All 4D flow CMR datasets were prospectively acquired in patients with BAV (n = 48) and healthy subjects (n = 25). Of the 48 patients, 23 (48%) patients had never had an aortic coarctation (nor repair thereof), whereas the remaining 25 patients previously underwent surgical repair for aortic coarctation. The healthy subjects were recruited based on the absence of a history of cardiovascular disease. All datasets [5] as well as a subgroup of patients with repaired coarctation and healthy subjects [29] were previously used for the evaluation of abnormal magnitude of hemodynamics. Furthermore, a subgroup of the healthy subjects was used to evaluate abnormal hemodynamics in patients with transcatheter aortic valve repair and implantation of a bileaflet mechanical valve [30, 31]. All participants provided written informed consent. The study protocol was approved by the local Institutional Review Board.

### CMR imaging

All BAV subjects underwent a CMR exam including contrast-enhanced (CE) CMR angiography and 4D flow CMR of the aorta. All healthy subjects underwent 4D flow CMR without contrast. All scans were carried out on at 3 T (Ingenia, Philips Healthcare, Best, The Netherlands). The scan parameters for all 4D flow CMR datasets were: spatial resolution of 2.5 mm × 2.5 mm × 2.5 mm, temporal resolution: approximately 42 ms depending on heart rate (24 cardiac phases) with TE/TR/flip angle of 2.1 ms/3.4 ms/8°. Velocity encoding (VENC) was set to a uniform (identical in three directions) 150–250 cm/s based on a through-plane flow CMR scout. Electrocardiographic gating was applied for keeping track of the RR-interval. A respiratory navigator was placed on the lung-liver interface for data acquisition in the end-expiration phase. All scans were accelerated with k-t PCA acceleration with a factor of 8 [32], resulting in a scan time of approximately 8 min.

### Data analysis

Forty-seven patients underwent echocardiography for the assessment of aortic stenosis (AS) and aortic regurgitation (AR). AR was defined as non, mild, moderate and severe based on the vena contracta width, regurgitation jet width relative to left ventricular outflow tract  diameter, pressure halftime and the presence of diastolic flow reversal in the descending aorta. AS was defined as none, mild, moderate and severe when peak velocity was ≤ 2.5 ms, 2.6–2.9 m/s, 3.0–4.0 m/s, and > 4 m/s respectively [33].

For 47 patients a CE-CMR angiogram was available on which the aortic diameter at the level of the sinotubular junction (STJ) and the mid-ascending aorta (mAA) was measured by a cardiovascular radiologist with over 15 years of clinical experience (R. N. P.). The mAA diameter was defined as the maximum length of two lines drawn perpendicular to the aortic wall at the level of the main pulmonary artery [34]. Furthermore, weight, blood pressure and the presence of hypertension were assessed.

The 4D flow CMR datasets were reconstructed using MRecon (Gyrotools, Zurich, Switzerland) with algorithms that included correction for background phase offsets caused by concomitant fields and eddy currents by fitting a plane through stationary tissue and subtract the fitted values from the image. Phase contrast (PC) CMR angiograms were created by multiplying the PC magnitude images with the absolute velocity images, followed by averaging over all cardiac time frames. The peak systolic velocity vectors in the aortas were subsequently masked using semi-automatically (thresholding, manually erasing surroundings, watersheds, filling holes and smoothing) created segmentations from the PC-CMR angiograms in Mimics (Materialise, Leuven, Belgium), a method that has shown good reproducibility [35]. Peak systole was defined as the cardiac phase with the highest velocity averaged over all voxels in the segmentation. The isosurface of the segmentation was subsequently meshed to automatically define coordinates on the aortic wall (Matlab, Mathworks, Natick, Massachusetts, USA). To obtain a relatively smooth surface without losing valuable information about structure, the surface was smoothed with a Laplacian filter [36]. On each point on the wall, WSS was calculated by fitting splines from zero velocity at the vessel wall through the velocity values at two equidistant points along the normal vector placed at half the local radius and at the local radius of the aortic lumen [37]. The viscosity used for WSS calculation was 3.2cP.

The 4D flow CMR data were further analysed in Cardiovascular Angiographic Analysis Systems MR Flow software (Pie Medical Imaging BV, Maastricht, The Netherlands): peak systolic streamlines were emitted from a plane perpendicular to the aorta at the level of the STJ and at the level of the mAA. The streamlines were scored for helical and vortical flow by two clinical experts (R. N. P. with 15 years and S. M. B. with 10 years of experience in cardiovascular imaging) similar to the protocol described by Burk et al. [12] and Knobelsdorff et al. [13]. Helical flow was considered a regional fluid circulation around an axis parallel to bulk fluid motion (i.e. along the longitudinal axis of the vessel), thereby creating a corkscrew-like motion. Vortical flow formation was defined as revolving streamlines around a point with a rotation direction of more than 90° from the physiological flow directions. The streamlines were attributed a score of 0 (= no or little helicity/vorticity, flow rotation < 180°), 1 (= moderate helicity/vorticity, flow rotation < 360°) or 2 (= severe helicity/ vorticity, flow rotation > 360°) [12]. After initial reading, the observers were given the opportunity to adapt their scores in mutual consultation.

### Local normalized helicity and vorticity

Local normalized helicity and vorticity and of the aortic velocity fields were calculated by methods previously described by Garcia et al. [21]. The vorticity $\omega$ was calculated by $\omega ={\text{curl}} V$ using a fourth-order Richardson scheme where $V$ is the 3D velocity field. Local normalized helicity (LNH) was calculated using $LNH=\frac{V\cdot \omega }{\left|V\right|\left|\omega \right|}$. The volume of absolute LNH > 0.6 and absolute vorticity were used for quantification purposes.

### Cohort-averaging of normal volunteers

A shared geometry of the healthy subjects was created by rigid co-registration of the aortic segmentations followed by determination of the 3D shape that showed the maximum overlap of the aortas [38]. Next, each normal aorta was registered to the shared geometry by affine registration in order to maximize the similarity between the shared geometry and the registered aorta. The velocity and WSS vectors were transformed by only the rotational part of the affine transformation in order to prevent scaling of the vectors. The rotational part of the affine transformation matrix was obtained by division by the scaling factor, which was calculated as the cubed determinant of the transformation matrix [39]. Now, the x,- y- and z-components of the vectors were interpolated (nearest neighbour) to the shared geometry, followed by averaging over the cohort.

### Subject-specific mapping of abnormally directed velocity and WSS

Similar to a previously published methodology, the normal-averaged velocity and WSS map were registered to the patient-specific aortic geometry followed by nearest neighbour interpolation of the x-, y- and z-components of the vectors [4]. The angle between the velocity and WSS vectors of the healthy-averaged maps and the patient-specific map calculation was calculated by $\mathrm{cos}\theta =\frac{u\cdot v}{\left|u\right|\left|v\right|},$ where $u$ and $v$ are the patient-specific and cohort-averaged velocity or WSS vector, respectively. Note that this expression results in $\theta$ ranging between 0° and 180°.

Next, the subject-specific velocity and WSS vectors that deviated > 120°, between 120° and 60° and < 60° from the healthy-averaged map were coloured in red, yellow and green respectively. Using in-house developed Matlab (Mathworks) software, the ascending aorta was isolated by dividing lines drawn orthogonal to the approximate level of the STJ and proximal to the brachiocephalic trunk and further subdivided into an inner and outer curvature, defined as the anterior respectively posterior half of the aorta [40]. Moreover, the proximal descending aorta was isolated by delineation from the left subclavian artery to the descending aorta at the level of pulmonary artery. See Fig. 2 for visualization of region separation. In these regions the volume of abnormally directed velocity (in cm^3^) and the surface of abnormally directed WSS (in cm^2^) was quantified.

### Ensemble mapping of abnormally directed velocity and WSS

Analogous to the creation of a shared geometry for the healthy subjects, a shared geometry was created for the BAV subjects. All subject-specific abnormally directed velocity and WSS maps were subsequently registered and interpolated to the shared geometry. By summing the interpolated subject-specific maps, a map was created that shows the incidence (expressed as a percentage of the number of subjects) and the location of abnormally directed velocity and WSS [5].

### Statistical analysis

Differences in age and gender between the patients and healthy subjects were tested with a Wilcoxon rank sum test and a Fisher test, respectively. Other differences in patient characteristics between the patients with and without repaired coarctation were tested with a Wilcoxon rank sum test as well. Differences in abnormally directed velocity volume and WSS surface between the inner and outer ascending aortic curvature and between the BAV patients and BAV patients with repaired coarctation in these regions and the descending aorta were tested with a Wilcoxon rank sum test. Agreement and differences between observer scorings were quantified with the intra-class correlation coefficient and the Wilcoxon rank sum test. Per observer the qualitative helicity and vorticity scores were correlated with the abnormally directed velocity volumes and WSS surfaces using linear regression with correlation coefficient R being reported.

Furthermore, total multiple linear regression models were investigated for the abnormally directed velocity volumes and WSS surfaces with age, gender, weight, blood pressure, presence of hypertension, STJ diameter, mAA diameter, echo-derived peak velocity and echo-derived AR severity with correlation coefficient R^2^ being reported. Single regression with these parameters was performed as well with correlation coefficient R being reported. The variables included in the model were tested for collinearity using a variance inflation factor of > 10 as a threshold to identify highly collinear variables [41]. Since the statistical analyses contain four attributes, namely velocity volume and WSS surface deviating between 120° and 60° and > 120°, Bonferroni correction was performed and P < 0.0125 (P < 0.05/4) was considered significant for all statistics.

## Results

The characteristics of the patients are summarized in Table 1. The BAV patients without repaired coarctation (BAV−CoA) were older, had higher peak velocity and significantly larger aortas than the BAV+CoA patients.

**Table 1:** Characteristics of bicuspid aortic valve (BAV) patients without repaired coarctation (BAV−CoA) and BAV patients with repaired coarctation (BAV + CoA)

	BAV−CoA (n = 23)	BAV+CoA (n = 25)	P*	All (n = 48)
Age (years) range	42 ± 14	34 ± 9	**0.041**	38 ± 12
Gender [male/female]	14/9	16/9	1.000	28 /18
Weight [kg]	78 ± 13	76 ± 11	0.535	77 ± 12
Blood pressure [mmHg]	130 ± 15^a^/80 ± 9^a^	135 ± 19/80 ± 11	0.573/0.717	133 ± 17^b^/80 ± 10^b^
Hypertension [present/absent]	6/16^a^	11/14	0.067	17/30^b^
Peak velocity echo (m/s)	2.4 ± 1.0	1.9 ± 0.6^c^	**0.047**	2.2 ± 0.8^d^
Aortic valve stenosis echo None/mild/moderate/severe	16/2/3/2	21/2/1/0^c^	0.108	37/4/4/2^d^
Aortic valve regurgitation echo None/mild/moderate/severe	5/10/7/1	7/13/4/0^c^	0.214	12/23/11/1^d^
Sinotubular junction diameter (mm)	35.0 ± 5.5	30.0 ± 4.6^e^	**0.005**	32 ± 5.5^f^
Mid-ascending aortic diameter (mm)	41.0 ± 6.5	33.0 ± 4.4^e^	** < 0.001**	37 ± 6.8^f^

Bold values are statistically significant

*Wilcoxon rank sum test, significance at P < 0.05

^a^n = 22, no blood pressure measurement for one subject

^b^n = 47, no blood pressure measurement for one subject

^c^n = 24, no echocardiography for one subject

^d^n = 47, no echocardiography for one subject

^e^n = 24, no CE-CMR angiogram for one subject

^f^n = 47, no CE-CMR angiogram for one subject

The age of the healthy subjects was 37 ± 13 years which was not significantly different from all patients (Wilcoxon rank sum test, P = 0.67). The gender distribution of the healthy subjects was 15 men: 10 women, which was similar to the BAV patients (Fisher exact test, P = 0.63).

**Fig. 1:**
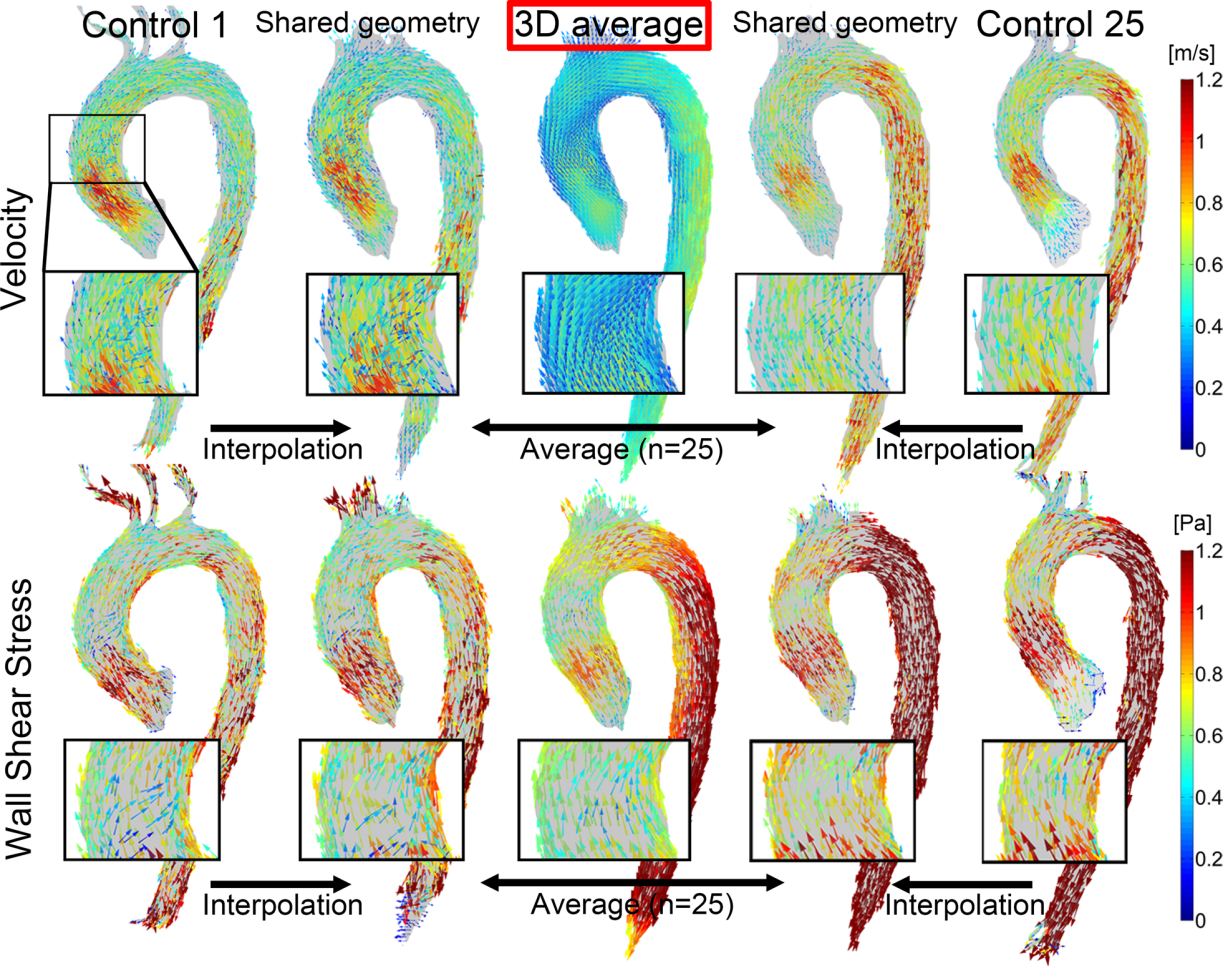
After interpolation of the velocity vectors (top row) and wall shear stress (WSS) vectors (bottom row) of the healthy subject to the shared geometry, the vectors are averaged resulting in a cohort-averaged velocity and WSS vector map

In Fig. 1 the cohort-averaged normal velocity and WSS vector maps are displayed. The velocity and WSS vectors in the ascending aorta were directed along the aortic curvature. Aortic segmentation took approximately 30 min and creation of a normal shared geometry as well. Peak systolic WSS calculation took approximately 5 min and creating an normal atlas as well.

**Fig. 2:**
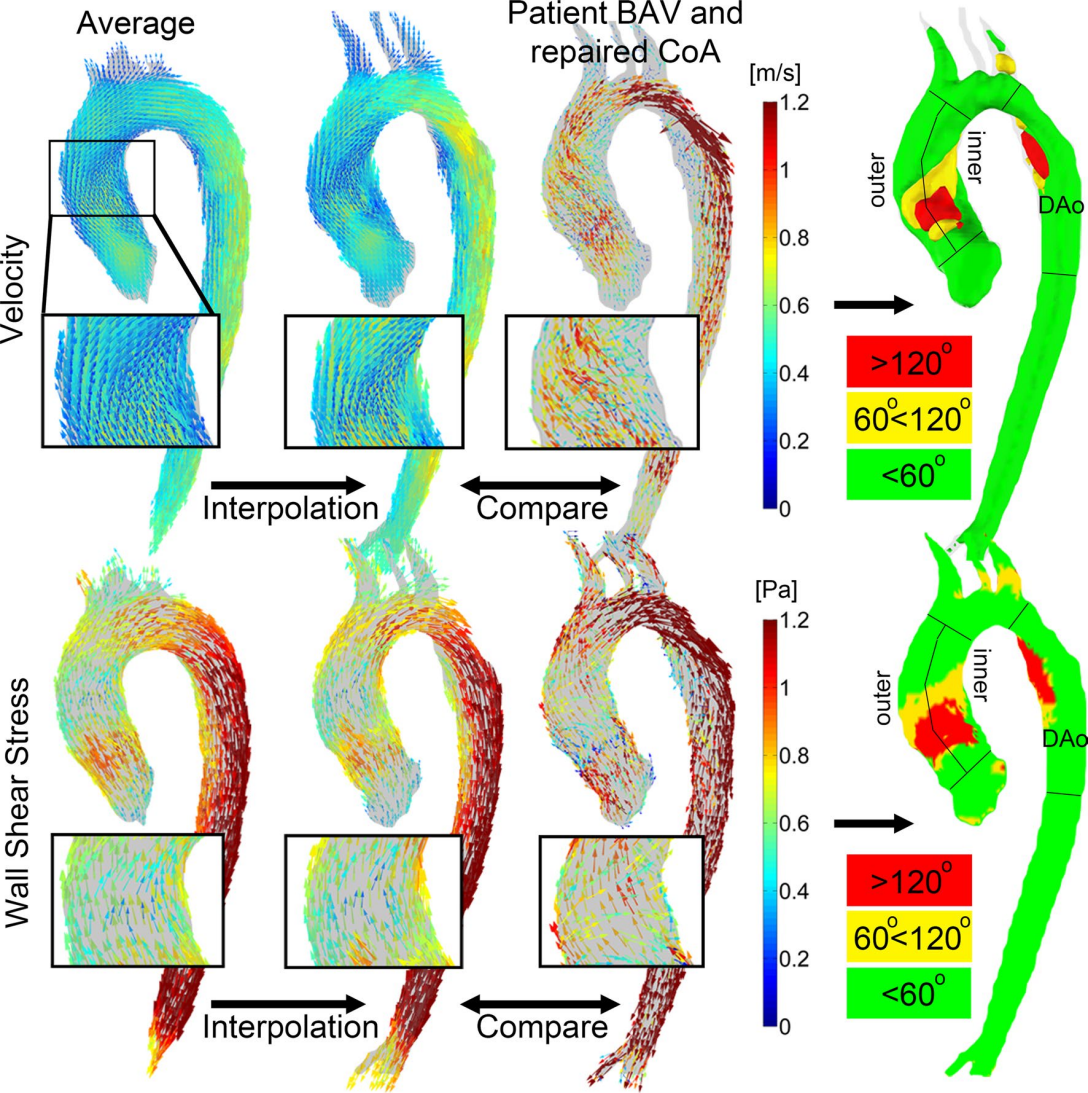
After interpolation of the velocity vectors (top row) and WSS vectors (bottom row) of the cohort-averaged normal map to the geometry of an example subject with bicuspid aortic valve (BAV) and repaired coarctation (CoA), a comparison is performed between the velocity and WSS vectors of the BAV subject with the interpolated cohort-averaged normal map. Regions where the velocity and WSS vector deviates more than 120° are colored in red, more than 60° but less than 120° in yellow, and less than 60° in green. The abnormally directed velocity volume and WSS surface is quantified in the inner and outer ascending aorta and the descending aorta (DAo)

In Fig. 2 the workflow to create abnormal direction velocity (top) and WSS (bottom) maps is displayed for one example BAV+CoA patient. Abnormally directed blood flow velocity and WSS was present at the proximal ascending aorta and at the descending aorta at the level of the repaired coarctation. Abnormally directed hemodynamic maps are created within one minute.

In Table 2 the average volume of velocity and surface of WSS vectors deviating between 60° and 120° and more than 120° compared to the healthy subjects are given for the BAV−CoA patients, the BAV+CoA patients and both BAV cohorts combined for the inner curvature, the outer curvature, the combined inner and outer ascending aorta and the proximal descending aorta. The BAV−CoA patients had more abnormal velocity and WSS direction than the BAV+CoA patients, except in the descending aorta, where the abnormal direction of hemodynamics was higher in the BAV+CoA patients than BAV−CoA patients. For all patients combined, the volume and surface of abnormally directed hemodynamics > 120° was higher in the inner ascending aorta curvature than the outer curvature.

**Table 2:** Abnormally directed velocity volumes and wall shear stress (WSS) surfaces compared between BAV patients with and without repaired coarctation (BAV+CoA and BAV−CoA, respectively) in the inner and outer ascending aorta, the entire ascending aorta and the descending aorta

		Velocity volume (cm^3^)					WSS surface (cm^2^)				
		Inner asc aorta	Outer asc aorta	P*	Ascending aorta	Descending aorta	Inner asc aorta	Outer asc aorta	P*	Ascending aorta	Descending aorta
60° > % < 120°	BAV−CoA	12.7 ± 7.9	12.0 ± 11.0	0.455	24.6 ± 18.4	3.3 ± 3.8	10.2 ± 5.6	9.1 ± 8.0	0.210	19.3 ± 12.3	4.4 ± 4.8
	BAV+CoA	5.5 ± 3.9	3.4 ± 2.3	0.074	8.9 ± 5.8	8.7 ± 8.3	6.3 ± 4.5	3.8 ± 2.5	0.063	10.1 ± 6.6	12.2 ± 11.5
	P*	** < 0.001**	** < 0.001**	–	** < 0.001**	**0.002**	0.021	** < 0.001**	–	**0.004**	** < 0.001**
	All	8.9 ± 7.1	7.6 ± 8.9	0.136	16.4 ± 15.4	6.1 ± 7.0	8.2 ± 5.4	6.3 ± 6.3	0.033	14.5 ± 10.7	8.4 ± 9.7
% > 120°	BAV−CoA	8.7 ± 6.8	4.9 ± 5.3	0.022	13.5 ± 9.1	0.7 ± 0.9	9.4 ± 5.8	7.0 ± 6.2	0.053	16.3 ± 8.5	1.6 ± 1.4
	BAV+CoA	3.1 ± 2.8	1.1 ± 1.0	**0.003**	4.1 ± 3.3	1.6 ± 1.2	4.6 ± 3.5	2.4 ± 2.6	0.013	6.9 ± 5.2	3.3 ± 2.6
	P*	**0.001**	** < 0.001**	–	** < 0.001**	0.024	**0.002**	** < 0.001**	–	** < 0.001**	**0.009**
	All	5.8 ± 5.8	2.9 ± 4.2	**0.001**	8.6 ± 8.2	1.2 ± 1.2	6.9 ± 5.3	4.6 ± 5.2	**0.008**	11.4 ± 8.4	2.5 ± 2.2

Bold values are statistically significant

*Wilcoxon rank sum test, P < 0.0125 significant

### Helicity and vorticity scoring compared to abnormal direction

**Fig. 3:**
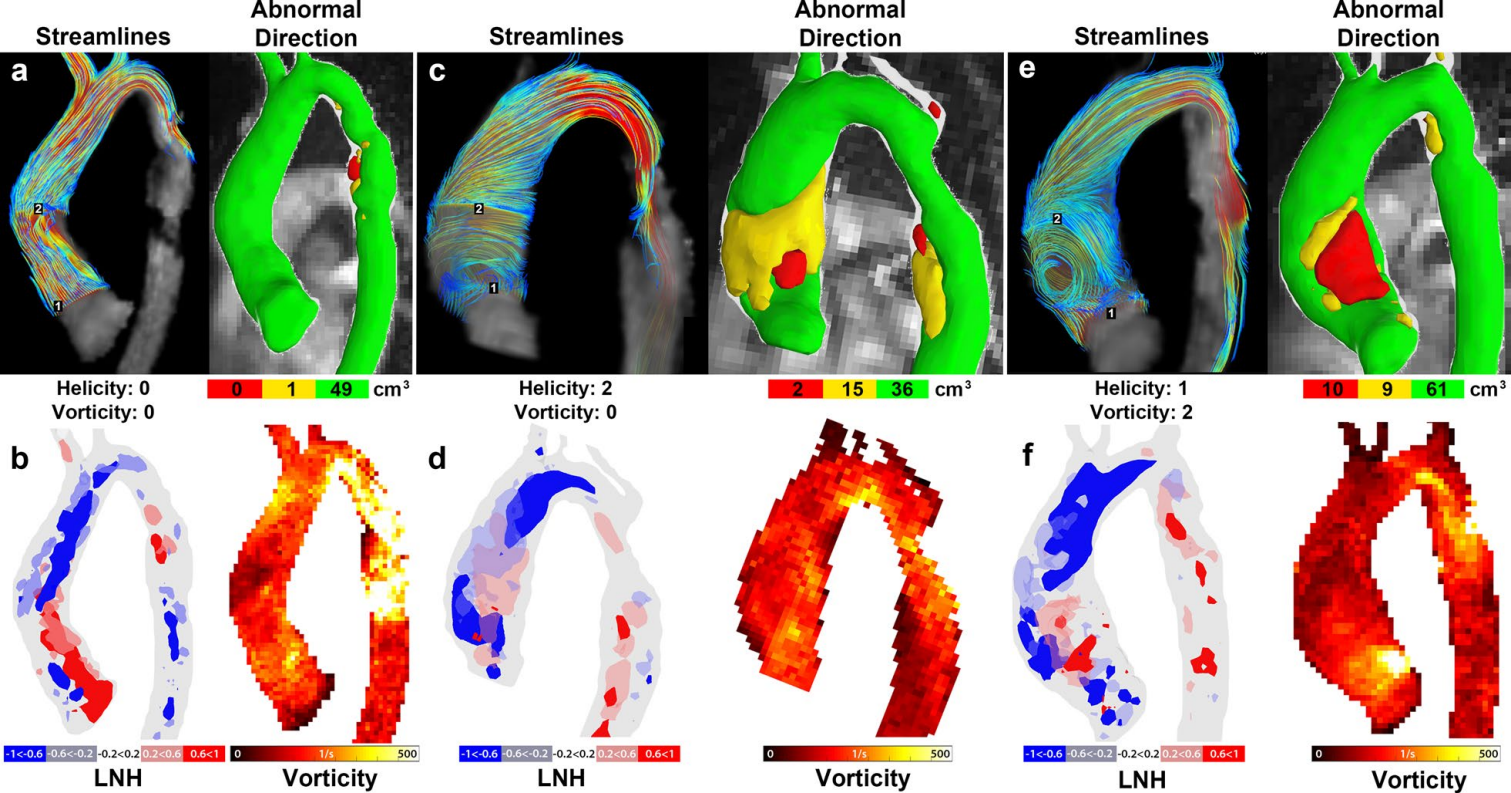
**a**, **c** and **d** streamlines, abnormal direction maps [laid over a maximum intensity projection of the phase contrast (PC) CMR angiogram A] in three patients with BAV and repaired coarctation, and **b**, **d** and **f** corresponding local normalized helicity (LNH) maps and vorticity maximum intensity projections (MIPs)

In Fig. 3a, c, e, three examples are shown of streamline maps with helicity and vorticity scores (equal by both observers) compared to abnormal velocity direction maps with corresponding volumes in BAV+CoA patients. In Fig. 3a, it can be seen that a patient with a helical and vortical score of 0 had only 1 cm^3^ of abnormally directed velocity of between 60° and 120° in the ascending aorta. In Fig. 3c a patient with the highest helicity score showed 15 cm^3^ volume of abnormally directed velocity of between 60° and 120° and in Fig. 3d a patient with a vortex in the proximal aorta had 10 cm^3^ of abnormally directed velocity > 120°. Note that all three subjects had abnormally directed velocity in the proximal descending aorta at the level of the repaired aortic coarctation. The ICC for observer scoring was 0.62 for helicity and 0.58 for vorticity. Between observers, a significant difference was found for the vorticity scorings (Wilcoxon rank sum test, P = 0.002) in contrast to the helicity scores (P = 0.23).

In Table 3 the correlation coefficients and P-values for linear regression between the helicity and vorticity scores of both observers and the abnormally directed velocity volumes and WSS surfaces in the ascending aorta are given. For both observers, moderate correlations were found for vorticity scores with abnormally directed velocity and WSS of > 120°.

**Table 3:** Regression coefficients and P value maps between abnormally directed velocity and WSS and observer scoring of helicity and vorticity

Score		Helicity		Vorticity	
		R	P*	R	P*
Velocity (cm^3^)					
Observer 1	60° < % < 120°	**0.49**	** < 0.001**	0.19	0.194
	% > 120°	**0.37**	**0.011**	**0.47**	** < 0.001**
Observer 2	60° < % < 120°	0.23	0.110	0.21	0.151
	% > 120°	0.04	0.766	**0.49**	** < 0.001**
WSS (cm^2^)					
Observer 1	60° < % < 120°	**0.59**	** < 0.001**	0.18	0.287
	% > 120°	**0.36**	**0.012**	**0.55**	** < 0.001**
Observer 2	60° < % < 120°	0.36	0.013	0.08	0.581
	% > 120°	0.11	0.461	**0.54**	** < 0.001**

Bold values are statistically significant

*Linear regression with P < 0.0125 considered significant

### Local normalized helicity and vorticity scoring compared to scorings and abnormal direction

In Fig. 3b, d, f, three examples are shown of LNH and vorticity maps in BAV+CoA patients. All three patients had large regions of high negative LNH in the distal ascending aorta. Only the patient with the zero helicity and vorticity scores had a large region of high positive LNH in the proximal ascending aorta (Fig. 3b). The vorticity maps were similar for all three patients, except in the descending aorta, where the patient shown in b had higher vorticity than the others. The highest vorticity of the patient shown in f was located in the inner curvature of the aorta whereas the flow vortex seen in e was located near the outer curvature.

Additional file 1: Table S1 shows that, except in the descending aorta, the BAV−CoA patients had more LNH than patients with BAV+CoA, that there was more LNH in the inner than the outer aorta for all patients, and that LNH correlated well with abnormally directed velocity between > 60° and < 120°. Additional file 1: Table S1 also shows that quantitative vorticity did not differ between BAV+CoA positive and BAV−CoA patients, and also did not differ between ascending aortic regions. Higher vorticity was found in the descending aorta for BAV+CoA patients. Quantitative vorticity did not correlate with abnormally directed velocity > 120°.

### Relation of abnormal hemodynamic direction with conventional metrics

The characteristic parameters were tested for collinearity where the highest variance inflation factor was 2.6 for STJ diameter and mAA diameter. All parameters reported in Table 1 were thus included in the model. In Table 4 the results are given for the linear regression analysis between abnormally directed velocity volumes and WSS surfaces with a deviation of > 60° and < 120°, LNH and the patient characteristic parameters. STJ and mAA diameter were found to be independent predictors for abnormally directed velocity and WSS and LNH. Additionally, age was an independent predictor for LNH in the outer curvature of the aorta.

**Table 4:** Regression analyses between abnormally directed hemodynamics > 60°, < 120° and BAV subject characteristics

	Total model^a^		Age^b^ (years)		Gender^b^ (–)		STJ Diameter^c^ (cm)		mAA Diameter^c^ (cm)		Peak velocity Echo^c^ (m/s)		AR^c^ (–)	
	R^2^	P	R	P	R	P	R	P	R	P	R	P	R	P
Inner velocity (cm^3^)	**0.78**	** < 0.001**	0.32	0.025	0.15	0.311	**0.72**	** < 0.001**	**0.77**	** < 0.001**	0.33	0.022	0.27	0.064
Outer velocity (cm^3^)	**0.66**	** < 0.001**	0.32	0.028	0.05	0.743	**0.67**	** < 0.001**	**0.64**	** < 0.001**	0.30	0.043	0.21	0.162
Inner WSS (cm^2^)	**0.54**	** < 0.001**	0.13	0.388	0.11	0.456	**0.49**	** < 0.001**	**0.57**	** < 0.001**	0.33	0.023	0.17	0.356
Outer WSS (cm^2^)	**0.59**	** < 0.001**	0.24	0.099	− 0.02	0.916	**0.60**	** < 0.001**	**0.53**	** < 0.001**	0.29	0.051	0.16	0.282
Inner LNH (cm^3^)	**0.66**	** < 0.001**	0.33	0.022	0.09	0.538	**0.60**	** < 0.001**	**0.73**	** < 0.001**	0.19	0.198	0.22	0.120
Outer LNH (cm^3^)	**0.81**	** < 0.001**	**0.42**	**0.003**	0.18	0.222	**0.65**	** < 0.001**	**0.80**	** < 0.001**	0.35	0.016	0.20	0.187

Bold values are statistically significant

P < 0.0125 considered significant

*WSS* wall shear stress, *STJ* sinotubular junction, *mAA* mid-ascending aorta, *AR* aortic regurgitation; *LNH* local normalized helicity

^a^46 subjects

^b^48 subjects

^c^47subjects

In Table 5 the results are given for the linear regression analysis for abnormally directed velocity and WSS with a deviation > 120° and for quantitative vorticity. STJ and MAA diameter were found to be independent predictors for abnormally directed velocity and WSS. For abnormally directed hemodynamics in the inner curvature of the aorta, age and peak velocity were independent predictors as well. For WSS in the outer curvature, aortic insufficiency was an independent predictor. For quantitative vorticity peak velocity was an independent predictor. Weight, blood pressure and hypertension were not independent predictors for any variable. The single regression analysis for these parameters is therefore not reported in Tables 4 and 5.

**Table 5:** Regression analyses between abnormally directed hemodynamics > 120° and BAV subject characteristics

	Total model^a^		Age^b^ (years)		Gender^b^ (–)		STJ Diameter^c^ (cm)		MAA Diameter^c^ (cm)		Peak velocity Echo^c^ (m/s)		AR^c^ (–)	
	R^2^	P	R	P	R	P	R	P	R	P	R	P	R	P
Inner velocity (cm^3^)	**0.71**	** < 0.001**	**0.59**	** < 0.001**	**0.40**	**0.005**	**0.51**	** < 0.001**	**0.68**	** < 0.001**	**0.45**	**0.001**	0.15	0.302
Outer velocity (cm^3^)	**0.64**	** < 0.001**	0.28	0.052	0.08	0.585	**0.59**	** < 0.001**	**0.64**	** < 0.001**	0.32	0.031	0.33	0.023
Inner WSS (cm^2^)	**0.67**	** < 0.001**	**0.56**	** < 0.001**	0.33	0.023	**0.49**	** < 0.001**	**0.60**	** < 0.001**	**0.51**	** < 0.001**	0.11	0.484
Outer WSS (cm^2^)	**0.65**	** < 0.001**	0.23	0.105	0.09	0.529	**0.54**	** < 0.001**	**0.56**	** < 0.001**	0.30	0.038	**0.39**	**0.007**
Inner Vorticity (1/s)	**0.51**	**0.001**	− 0.34	0.018	− 0.09	0.546	− 0.12	0.428	− 0.24	0.103	**0.42**	**0.003**	0.15	0.302
Outer Vorticity (1/s)	**0.67**	** < 0.001**	− 0.21	0.158	0.06	0.703	− 0.15	0.305	− 0.21	0.159	**0.62**	** < 0.001**	0.19	0.199

Bold values are statistically significant

P < 0.0125 considered significant

*WSS* wall shear stress, *STJ* sinotubular junction, *MAA* mid-ascending aorta, *AR* aortic insufficiency

^a^46 subjects

^b^48 subjects

^c^47subjects

### Ensemble mapping of abnormally directed velocity and WSS

**Fig. 4:**
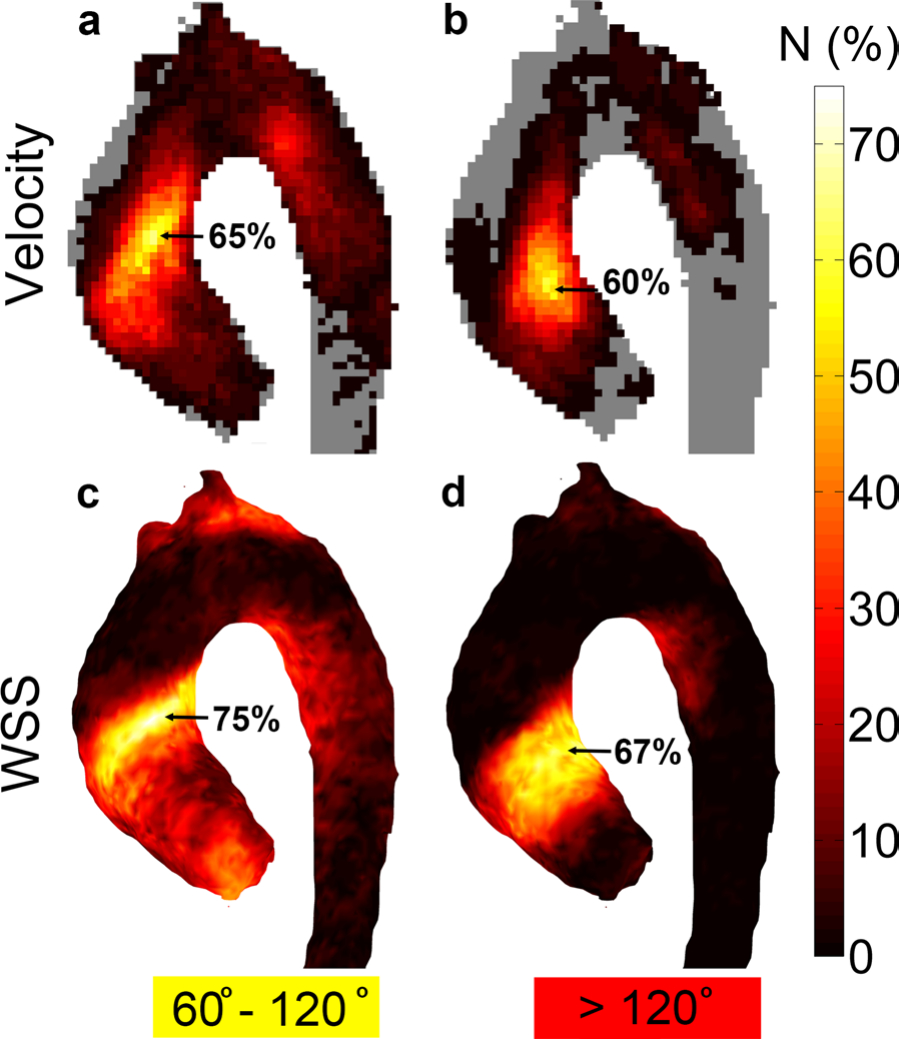
Ensemble maps of abnormally directed **a** velocity and **c** WSS deviating between 60° and 120° and **b** velocity and **d** WSS deviating more than 120°

In Fig. 4 the ensemble maps of abnormally directed velocity (top row) and WSS (bottom row) are displayed for vectors of the BAV patients deviating between 60° and 120° (left column) and more than 120° (right column) from normal volunteers. Creation of the ensemble maps took 20 min. At the level of the mAA, 65% and 75% of all patients had abnormally directed velocity and WSS, respectively, with a deviation between 60° and 120°. Slightly more proximal to the mAA, 60% and 67% had abnormally directed velocity and WSS, respectively, deviating more than 120°.

## Discussion

In this study a novel technology was presented to visualize and quantify peak systolic abnormally directed velocity and WSS in the aorta by comparing BAV patients with healthy subjects. We found (1) that vorticity scores of both observers correlated moderately with abnormally directed velocity and WSS of > 120°, (2) that more abnormally directed velocity and WSS was found in the descending aorta of BAV+CoA than BAV−CoA patients, (3) that abnormally directed velocity between 60° and 120° correlated moderately with LNH but that abnormally directed velocity > 120° did not correlate with quantitative vorticity, and (4) that abnormally directed velocity between 60° and 120° and < 120° correlated moderately with aortic diameters and with age and peak velocity (as a measure for stenosis). In the investigated cohort more than 60% of participants had abnormally directed velocity and WSS, mainly located at the aortic inner curvature at the level of the mAA.

Historically, streamline or pathline scoring of helicity and vorticity has been performed to relate abnormal flow patterns to aortic dilation [12, 13]. Scoring of images is inherently operator-biased, which is supported by the moderate ICCs and significant difference between observers found in the vorticity scores. Still, the significant correlations found between abnormally directed hemodynamics and the scoring of peak systolic streamline patterns indicate that the (arbitrarily chosen) threshold of > 120° may be a robust descriptor for vorticity. The methodology was also able to measure a larger amount of abnormally directed hemodynamics in the descending aorta of patients with repaired coarctation than without.

Several previous studies have investigated fully quantitative techniques to evaluate helicity and vorticity in aortic blood flows [21, 28]. The approach presented here differs from these studies in a sense that no mathematical equations describing helicity or vorticity need to be applied, but that potentially more intuitive 3D visualization and quantification is achieved by registration of a 3D averaged normal map to a patient dataset. Interestingly, abnormally directed velocity > 120° showed no agreement with quantitative vorticity. Vorticity is quantified by the curl of the vector field, which describes the rotation of the flow at a very local scale, i.e. per voxel. Quantitative voxel-wise vorticity analyses may not be sufficiently sensitive to visualize and quantify larger vortices, such as those occur along the entire width of dilated ascending aortas.

By comparing 3D voxel-based reversed flow maps to plane-based quantification of reversed flow, Shen et al. found an underestimation in plane-based measurements and underlined the importance of the 3D analysis [23]. We followed this reasoning by designing 3D methodology which, in the case of visualization of regions deviating more than 120° from healthy subjects (which can be considered regions of reversed flow), closely resembles the methodology presented by Shen et al. The added benefits of our approach are that also helical flow (deviating between 60° and 120° from healthy subjects) can be visualized and that the technology can be applied to WSS as well.

Studies investigating WSS direction in BAV disease mostly employed methodology to decompose the WSS vector in an axial and circumferential component [6, 17, 27]. The ensemble maps presented here show that abnormally directed WSS deviating more than 120° predominantly manifests at the inner curvature of the aorta, which should correspond with a negative axial WSS in this region. Yet, the negative axial WSS was either not reported or not presented as a major finding in the previous studies. One potential reason is that the inner curvature was not deemed of interest because other studies have reported local aortic wall abnormalities at the more distal outer curvature of the aorta, where high WSS magnitude was found [7, 8]. It is therefore unlikely that WSS-mediated aortic wall degradation occurs at the proximal inner curvature in the region of reversed WSS. Nonetheless, we speculate that altered WSS direction at the inner curvature may be predictive for outward aortic remodelling. In addition, altered flow direction may contribute to systemic hypertension and premature atherosclerosis in adults with BAV+CoA. Consequentially, it may be important to track patients with these abnormalities.

Several studies have found an association between altered flow and WSS direction with aortic dilation employing 4D flow CMR in BAV disease [6, 17, 27]. One study suggested that characterization of blood flow patterns may help to select BAV patients for follow-up to monitor the risk for aortic dilation [42]. Our study contributes to this body of literature with an approach for characterization of abnormal velocity and WSS direction. Additionally, abnormally directed velocity and WSS was found at the level of the repaired coarctation, making the approach potentially suitable for post-surgery inspection of coarctation repair and monitoring BAV+CoA patients for recoarctation progression.

Altered flow patterns and decreased circumferential WSS have been reported in Marfan patients as well [22, 43]. The maps of abnormally directed hemodynamics may have added value in diagnosis, timing of surgery and patient monitoring in the Marfan population.

The main limitation of the study is that abnormally directed hemodynamics are detected in the peak systolic timeframe only. The reason is that 4D flow CMR data has limited signal in the diastolic timeframes preventing accurate segmentation that takes the motion of the aorta into account. A technique to obtain time-resolved segmentations has been developed in the past few years [44, 45], but this methodology may not work in cases of suboptimal image quality. Validated time-resolved segmentations will be important since the oscillatory shear index, i.e. the variability of WSS direction over time, is a marker for atherosclerosis and potentially other aortic diseases that requires accurate wall definition (with aortic motion) over all timeframes.

Another limitation is that arbitrarily chosen thresholds of 60° and 120° were employed. To define thresholds that may be more indicative for disease, longitudinal follow-up and patient outcome data are needed. This aim will be part of future work.

A final limitation is that risk factors for aortic dilation such as weight, race/ethnicity, blood pressure and smoking status were not recorded in the healthy subject cohort. The presence of these risk factors may have obscured some of the findings in the patient cohort. It is thus important to investigate these risk factors in the healthy population and create cohort-averaged normal maps stratified for these factors, which also permits more extensive investigation of risk factor associations with abnormally directed hemodynamics.

## Conclusions

In conclusion, a methodology was presented to map abnormal velocity and WSS direction at peak systole in the aorta of patients with BAV. Abnormal velocity and WSS direction correlated moderately with vorticity scores and with aortic diameter and peak velocity. Ensemble maps showed that in more than half of the patients, abnormal hemodynamic direction was present. The possibility to reveal these directional abnormalities in 3D provides a new tool for hemodynamic characterization in BAV disease.

## Supplementary Information

**Additional file 1: Table S1.** Quantification of LNH and vorticity with differences between regions and subjects, and correlations with qualitative scorings and abnormally directed velocity.

## Abbreviations

AR: Aortic regurgitation
AS: Aortic stenosis
BAV: Bicuspid aortic valve
BAV−CoA: Bicuspid aortic valve without coarctation
BAV+CoA: Bicuspid aortic valve with aortic coarctation
CE: Contrast enhanced
CMR: Cardiovascular magnetic resonance
LNH: Local normalized helicity
mAA: Mid-ascending aorta
PC: Phase contrast
STJ: Sinotubular junction
VENC: Velocity encoding
WSS: Wall shear stress

## Availability of data and material

All data is available on reasonable request.
