# Supplementary material for: Fully quantitative mapping of abnormal aortic velocity and wall shear stress direction in patients with bicuspid aortic valves and repaired coarctation using 4D flow cardiovascular magnetic resonance
Source: J Cardiovasc Magn Reson. 2021 Feb 15;23:9. doi: 10.1186/s12968-020-00703-2 (PMC7885343; doi:10.1186/s12968-020-00703-2)
Supplement: Supplementary file 1 — Additional file 1: Table S1. Quantification of LNH and vorticity with differences between regions and subjects, and correlations with qualitative scorings and abnormally directed velocity. [file 12968_2020_703_MOESM1_ESM.docx]

| *Table S1. Quantification of LNH and vorticity with differences between regions and subjects, and correlations with qualitative scorings and abnormally directed velocity* | | | | | | | | | | | | | | |
| --- | --- | --- | --- | --- | --- | --- | --- | --- | --- | --- | --- | --- | --- | --- |
|  |  | Average, standard deviation and significant differences | | | | | Correlations^2^ | | | | | | | |
|  |  |  |  |  |  |  | Comparison with abnormally directed velocity volume (cm^3^) | | | | | | | |
|  |  | Inner  asc aorta | Outer  asc aorta | P^1^ | Ascending  aorta | Descending  aorta | Inner  asc aorta | | Outer  asc aorta | | Ascending aorta | | Descending  aorta | |
|  |  |  |  |  |  |  | R | P | R | P | R | P | R | P |
| LHN  (-) | BAV-CoA  (n=23) | 7.7 ± 4.5 | 6.2 ± 3.5 | 0.292 | 13.8 ± 7.6 | 6.7 ± 3.5 | **0.66** | **<0.001** | **0.64** | **<0.001** | **0.66** | **<0.001** | **0.72** | **<0.001** |
|  | BAV+CoA  (n=25) | 4.0 ± 1.7 | 2.4 ± 1.8 | **<0.001** | 6.3 ± 3.0 | 6.1 ± 2.8 | 0.37 | 0.066 | **0.52** | **0.008** | **0.57** | **0.003** | 0.41 | 0.041 |
|  | P^1^ | **<0.001** | **<0.001** | **-** | **<0.001** | 0.606 |  |  |  |  |  |  |  |  |
|  | All  (n=48) | 5.8 ± 3.8 | 4.2 ± 3.3 | **0.009** | 9.9 ± 6.8 | 6.4 ± 3.1 | **0.71** | **<0.001** | **0.72** | **<0.001** | **0.75** | **<0.001** | **0.38** | **0.007** |
| Vorticity  (1/s) | BAV-CoA  (n=23) | 119 ± 37 | 123 ± 48 | 1.000 | 121 ± 41 | 101 ± 35 | -0.26 | 0.226 | -0.13 | 0.561 | -0.09 | 0.677 | 0.08 | 0.733 |
|  | BAV+CoA  (n=25) | 121 ± 31 | 119 ± 32 | 0.801 | 120 ± 30 | 144 ± 53 | 0.06 | 0.786 | 0.06 | 0.773 | 0.18 | 0.395 | -0.48 | 0.016 |
|  | P^1^ | 0.710 | 0.757 | - | 0.726 | **0.003** |  |  |  |  |  |  |  |  |
|  | All  (n=48) | 120 ± 34 | 121 ± 40 | 0.783 | 121 ± 36 | 124 ± 50 | -0.17 | 0.261 | -0.07 | 0.660 | -0.02 | 0.905 | -0.10 | 0.482 |
| *^1^Wilcoxon rank sum test,* ***P<0.0125 considered significant****, ^2^LHN was compared with abnormally directed velocity 60°<%<120° and vorticity with >120°* | | | | | | | | | | | | | | |
